# Fluid and flexible minds: Intelligence reflects synchrony in the brain’s intrinsic network architecture

**DOI:** 10.1162/NETN_a_00010

**Published:** 2017-06-01

**Authors:** Michael A. Ferguson, Jeffrey S. Anderson, R. Nathan Spreng

**Affiliations:** Laboratory of Brain and Cognition, Human Neuroscience Institute, Department of Human Development, Cornell University, Ithaca, NY, 14853; Departments of Bioengineering and Neuroradiology, University of Utah, Salt Lake City, UT, 84132

**Keywords:** intelligence, fMRI, resting state functional connectivity, machine learning, cognition

## Abstract

Human intelligence has been conceptualized as a complex system of dissociable cognitive processes, yet studies investigating the neural basis of intelligence have typically emphasized the contributions of discrete brain regions or, more recently, of specific networks of functionally connected regions. Here we take a broader, systems perspective in order to investigate whether intelligence is an emergent property of synchrony within the brain’s intrinsic network architecture. Using a large sample of resting-state fMRI and cognitive data (*n* = 830), we report that the synchrony of functional interactions within and across distributed brain networks reliably predicts fluid and flexible intellectual functioning. By adopting a whole-brain, systems-level approach, we were able to reliably predict individual differences in human intelligence by characterizing features of the brain’s intrinsic network architecture. These findings hold promise for the eventual development of neural markers to predict changes in intellectual function that are associated with neurodevelopment, normal aging, and brain disease.

## Author Summary

In our study, we aimed to understand how individual differences in intellectual functioning are reflected in the intrinsic network architecture of the human brain. We applied statistical methods, known as *spectral decompositions*, in order to identify individual differences in the synchronous patterns of spontaneous brain activity that reliably predict core aspects of human intelligence. The synchrony of brain activity at rest across multiple discrete neural networks demonstrated positive relationships with fluid intelligence. In contrast, global synchrony within the brain’s network architecture reliably, and inversely, predicted mental flexibility, a core facet of intellectual functioning. The multinetwork systems approach described here represents a methodological and conceptual extension of earlier efforts that related differences in intellectual ability to variations in specific brain regions, networks, or their interactions. Our findings suggest that the neural basis of complex, integrative cognitive functions can be most completely understood from the perspective of network neuroscience.

Mapping the neural substrates of human intelligence could better inform our understanding of how brain development influences lifespan development across multiple functional domains, both in health and in disease (Furnham, 2008; Gottfredson, 2004; Schutte, 2014). The concept of general intelligence—Spearman’s g, or simply “g”—was first postulated by Spearman at the turn of the previous century (Spearman, 1904). Tests for cognitive performance share ubiquitous positive correlations, suggesting that a common “intelligence” factor may explain individual differences across a range of cognitive domains. Over decades of inquiry, it has been demonstrated that individual differences in Spearman’s g indeed robustly predict performance on laboratory tests of cognitive function, as well as adaptive behavior in real-world contexts (Kuncel, Hezlett, & Ones, 2004; Spearman, 1927). More recently, with the advent of functional neuroimaging methods, investigating the neural basis of “g” has been an active area of inquiry (Deary, Penke, & Johnson, 2010; Duncan, 2005; Duncan et al., 2000; Schultz & Cole, 2016).

For decades, cognitive science has described intelligence as an emergent property of dissociable processes (Churchland, 1986; Gregory & Zangwill, 1987; Sternberg & Detterman, 1986). Yet efforts to identify neural markers of “g” have typically focused on individual brain regions (Duncan, 2005; Duncan et al., 2000; Gray, Chabris, & Braver, 2003) or specific networks of functionally connected brain regions (Cole, Yarkoni, Repovs, Anticevic, & Braver, 2012; Lee et al., 2006; Song et al., 2008). Frontoparietal networks have received the widest attention as a possible single-network solution to understanding the biological underpinnings of individual differences in intellectual ability (Jung & Haier, 2007; Langeslag et al., 2013). Early efforts to characterize the neural basis of cognitive functioning, which assumed a one-to-one brain–behavioral mapping, have been increasingly challenged by models of whole-brain network interactions, particularly with respect to complex, integrative cognitive capacities such as intelligence (Bullmore et al., 2009; McIntosh, 1999; Sporns, Chialvo, Kaiser, & Hilgetag, 2004; van den Heuvel, Stam, Kahn, & Hulshoff Pol, 2009). This suggests that a systems-level, network-based approach may prove fruitful in identifying reliable neural markers of individual differences in human intellectual function.

Early localization studies investigated brain–behavior relationships in circumscribed brain regions because methodological constraints, including low temporal and spatial resolution, as well restrictions in computational capacity necessitated a univariate analytical approach (Duncan, 2005; Duncan et al., 2000). These analyses identified specific brain regions where the magnitude of activity varied as a function of performance on intelligence tasks (Duncan et al., 2000). Advances in fMRI data acquisition and analytical methods have dramatically improved the temporal and spatial resolution of neuroimaging protocols, enabling the measurement of regional interactions within distributed brain networks to characterize the neural architecture of intelligence (Lee et al., 2006; Smith et al., 2013).

More recently, studies have begun to characterize multinetwork dynamics, or the network architecture of the brain, as a neural marker of intelligence (Cole, Ito, & Braver, 2015). Properties of large-scale, distributed networks observed in the brain during a wakeful resting state have been identified as factors contributing to individual differences in intelligence (Finn et al., 2015; Hearne et al., 2016; Smith et al., 2015). Building from these earlier studies, here we apply spectral decompositions to resting-state functional connectivity (RSFC) from fMRI in order to derive estimates of the brain’s spatially overlapping functional architecture that may be sensitive to individual differences in “g.” Resting-state MRI is a powerful tool to detect and dissociate functional brain networks from patterns of interregional correlations in neuronal variability, estimated by variations in BOLD signal and measured in the absence of explicit task demands (Buckner, Krienen, Castellanos, Diaz, & Yeo, 2011; Fox et al., 2005). Previous studies have identified distributed contributions to intelligence in the brain by several methods, including the examination of pairwise connections without imposed network definitions (Hearne et al., 2016; Smith et al., 2015), or the exploration of functional relationships both with and without a priori network definitions (Finn et al., 2015). In these analyses, the functional correlations between pairwise regions represent the edges of distributed, spatially nonoverlapping networks (Finn et al., 2015; Hearne et al., 2016; Smith et al., 2015). Here we seek to identify a basis set of spatially overlapping functional networks in the resting brain and to determine the contributions of within-network and across-network interactivity to intelligence. We identified spatially overlapping network maps in an effort to characterize the one-to-many functional organization of the human brain that we believe is essential for understanding the relationships between cognition and neural systems (Bullmore et al., 2009; McIntosh, 1999; Sporns et al., 2004; van den Heuvel et al., 2009).

To identify a basis set of functional networks in the resting brain, we introduced the application of a multivariate statistical method, *spectral decomposition* (Alter, Brown, & Botstein, 2000; Yeung, Tegner, & Collins, 2002), to characterize an RSFC (i.e., intrinsic) network architecture in a large population of healthy young adults. This technique identifies the spectrum of spatially overlapping networks that covary across the duration of the resting-state fMRI scan. Early brain-imaging studies had applied spectral decompositions to task-based fMRI data (Bullmore et al., 1996; Friston, Frith, Fletcher, Liddle, & Frackowiak, 1996). To our knowledge, this is the first application of spectral analysis to RSFC data in order to describe the functional dynamics of intrinsic brain networks and predict individual differences in cognitive ability.

To evaluate whether individual differences in resting-state network dynamics may be a marker of intellectual functioning, we drew upon two measures that have previously been used to characterize intellectual capacity: fluid intelligence (i.e., cognitive control) and cognitive flexibility (Scott, 1962). In our study we investigated whether individual differences in these core facets of human intelligence are associated with differences in the intrinsic functional architecture of the brain, operationalized as the intrinsic synchrony or connectivity of functional networks. We predicted that greater synchrony within and among functional networks at rest would predict better performance on measures of fluid intelligence and of cognitive flexibility.

## RESULTS

### Spectral Decomposition for Multivariate Pattern Identification

**Figure 1.**
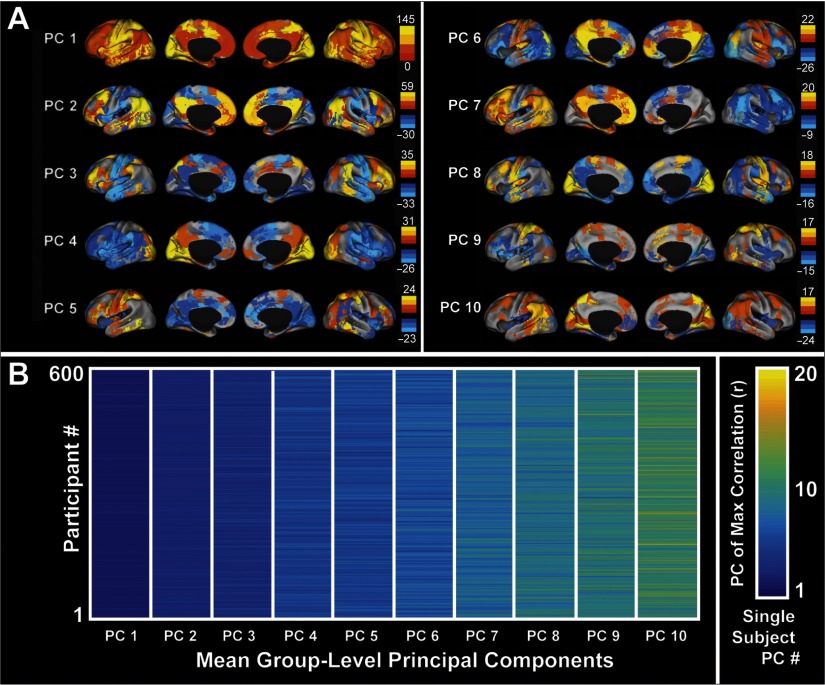
Principal components (PCs) of resting-state functional connectivity. (A) Top ten PCs (across-group FWE-corrected *p* < .05). Reliable positive and negative features are shown for each component. Color bars indicate *t*-values. (B) Correspondence of the most homologous single-subject PCs to the group-average PCs, shown for 600 subjects from the Human Connectome Project 900-subject release. The least variation across individuals exists in PC1, and variation across individual PC differences increases consecutively with PC order.

Spectral decomposition was used to determine the synchrony and structure of intrinsic brain networks. Spectral decompositions are a family of closely related analyses that describe the dominant components of a complex system. These include principal-component analysis (PCA), singular value decomposition, and eigendecompositions. Spectral decomposition identifies the spatial patterns of functional synchrony across brain regions, as well as their archi tecture or hierarchical organization, as determined by the synchrony strength, or persistent synchrony, of these networks. The resulting principal components (PCs) are ranked numerically according to their prominence within the resting-state architecture (Figure 1A). Heterogeneity in the subject-level PC topology was inversely related to singular values (i.e., PC rank); PC 1 was the most homogeneous across the population, and PC 10 the most heterogeneous (Figure 1B). The whole-brain networks described by RSFC PCs show features of well- characterized neurocognitive systems (Fox et al., 2005; Smith et al., 2009; Spreng, Sepulcre,Turner, Stevens, & Schacter, 2013; Yeo et al., 2011). For example, the group-mean RSFC components show synchrony of sensory and motor cortices (PC 1), the default network (PC 2), the salience network (ventral attention) relative to default regions (PC 3), the visual system (PC 4), and the dorsal attention network (PC 5). PC 6 comprises motor and default regions. PC 7 demonstrates a functionally independent mode of the left relative to right frontoparietal control network. PCs 8–10 represent frontoparietal functional ensembles, including posterior attention regions as aspects of canonical intrinsic networks (Chen et al., 2013).

All subcortical structures (thalamus, caudate, putamen, pallidum, hippocampus, amygdala, and nucleus accumbens) demonstrated bilateral positive associations with group-mean PCs 1 and 2. In both PCs 3 and 5, amygdala and hippocampus were functionally correlated and showed anticorrelation relative to all other subcortical structures. Subcortical functional associations between the hemispheres were largely symmetric across principal components, with the notable exception of PC 7. In PC 7, a left-lateralized subcortical configuration corresponded to left-lateralized patterns in cortical activity (Table 1).

**Table 1.** Principal-component (PC) subcortical associations

	****PC 1****	****PC 2****	****PC 3****	****PC 4****	****PC 5****	****PC 6****	****PC 7****	****PC 8****	****PC 9****	****PC 10****
Thalamus	L+ R+	L+ R+	L+ R+	L–	L– R–	L+ R+	L+	–	–	L– R–
Caudate	L+ R+	L+ R+	L+ R+	L– R–	L– R–	–	L+ R+	–	–	L– R–
Amygdala	L+ R+	L+ R+	L– R–	L–	L+ R+	–	L+	L– R–	–	L– R–
Hippocampus	L+ R+	L+ R+	L– R–	L+ R+	–	L+ R+	L+	L– R–	L+	L– R–
Pallidum	L+ R+	L+ R+	L+ R+	L–	L– R–	R+	L+	–	–	–
Nucleus accumbens	L+ R+	L+ R+	–	–	L– R–	L+ R+	L+	R–	–	–
Putamen	L+ R+	L+	L+ R+	L– R–	L– R–	–	L+ R+	–	–	L– R–

The table shows associations between PCs 1–10 and bilateral subcortical structures. Positive and negative functional relationships with left (L) and right (R) subcortical structures are shown only for significant regions.

### Eigenvalues and Behavior

**Figure 2.**
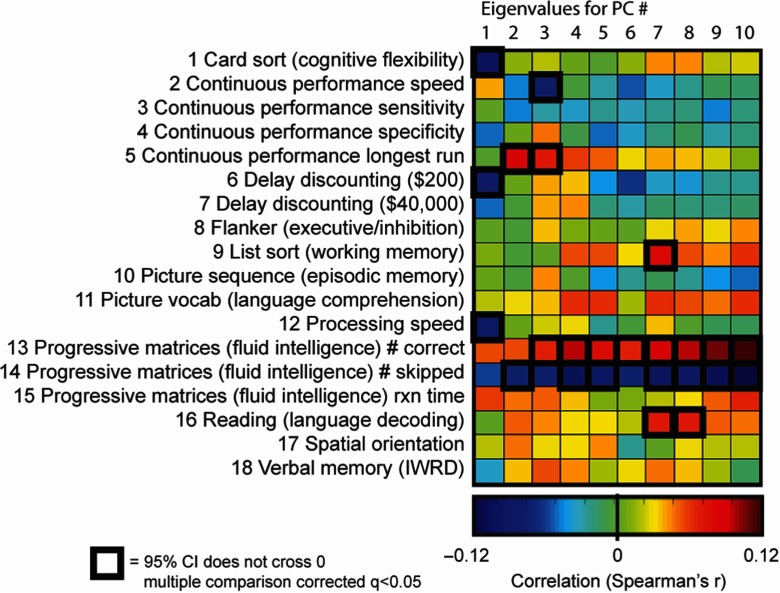
Cognitive measures and PC eigenvalues. Spearman correlations were calculated between cognitive performance measures and unscaled eigenvalues for PCs 1–10 (*n* = 600). Correlations with 95% confidence intervals that do not cross 0 and that survive multiple- comparison correction are highlighted. The cognitive measures demonstrating the broadest cor relations with PC eigenvalues are those for fluid intelligence. Because larger eigenvalues indicate stronger within-network synchrony, the results demonstrate that increased within-network synchrony for a broad range of PCs is positively correlated with fluid intelligence.

Correlation scores were calculated between cognitive measures and eigenvalues (i.e., measures of network synchrony) for PCs 1–10 (*n* = 600). PC eigenvalues demonstrated significant correlations with numerous measures of cognitive performance when corrected for multiple comparisons (Figure 2). As predicted, fluid intelligence demonstrated the greatest correlations with functional network eigenvalues. All correlations between eigenvalues (measures of within-network synchrony) and fluid intelligence were positive, indicating that greater within-network synchrony across a range of networks is predictive of fluid intelligence (means [95% CIs]: PC 3, *r* = 0.08 [0.015, 0.15]; PC 4, *r* = 0.13 [0.06, 0.19]; PC 5, *r* = 0.12 [0.05, 0.18]; PC 6, *r* = 0.09 [0.02, 0.15]; PC 7, *r* = 0.12 [0.06, 0.19]; PC 8, *r* = 0.14 [0.06, 0.21]; PC 9, *r* = 0.13 [0.07, 0.20]; PC 10, *r* = 0.13 [0.07, 0.20]; see Figure 2). Cognitive flexibility demonstrated a negative correlation with the eigenvalue of PC 1, indicating that greater global synchrony is associated with lower cognitive flexibility (*r* = –0.09, 95% CI [–0.16, –0.03]; Figure 2).

### Scaled Eigenvalues and Behavior

**Figure 3.**
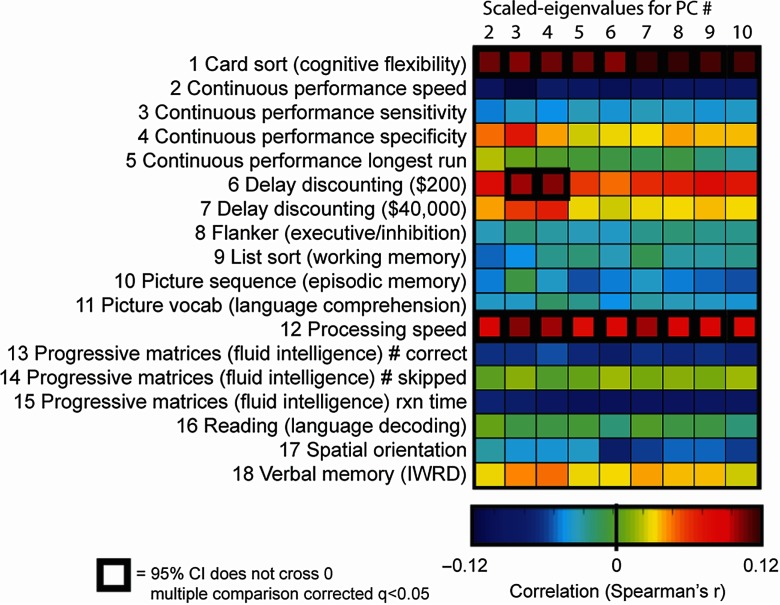
Cognitive measures and scaled eigenvalues for the PCs. Spearman correlations were calculated between cognitive performance measures and the scaled eigenvalues for PCs 2–10 (*n* = 600). Scaled eigenvalues were calculated by dividing each PC’s eigenvalue by the eigenvalue for PC 1 in each individual subject. Correlations with 95% confidence intervals that do not cross 0 and that survive multiple-comparison correction are highlighted. The cognitive measures demonstrating the broadest correlations with PCs are those for cognitive flexibility and processing speed. Larger scaled eigenvalues indicate stronger within-network synchrony relative to the global synchrony of an individual’s brain. Stronger within-network synchrony relative to the global synchrony (PC 1) is positively correlated with increased cognitive flexibility and more rapid processing speed across PCs 2–10.

To investigate the relationship between intellectual functioning and individual differences in network hierarchies, we also correlated the cognitive performance measures with the scaled eigenvalues for PCs 2–10. (Because the scaled eigenvalue for PC 1 is by definition equal to 1, PC 1 is not included in the scaled-eigenvalue comparisons.) Cognitive flexibility demonstrated positive correlations with each of the scaled eigenvalues for PCs 2 to 10 (means [95% CIs]: PC 2, *r* = 0.09 [0.03, 0.16]; PC 3, *r* = 0.09 [0.03, 0.16]; PC 4, *r* = 0.10 [0.03, 0.16]; PC 5, *r* = 0.09 [0.03, 0.17]; PC 6, *r* = 0.10 [0.03, 0.16]; PC 7, *r* = 0.10 [0.04, 0.17]; PC 8, *r* = 0.10 [0.04, 0.17]; PC 9, *r* = 0.10 [0.03, 0.17]; PC 10, *r* = 0.10 [0.03, 0.17]; Figure 3). This indicates that cognitive flexibility is supported by persistent synchrony across a broad array of functional networks. In contrast, as we identified above, global synchrony, or the baseline level of sustained synchrony across the whole brain, is associated with lower cognitive flexibility. A similar pattern was observed for processing speed, suggesting that both aspects of cognitive functioning are enhanced by greater synchrony in specific networks but negatively impacted by higher levels of global synchrony.

### Behavior/RSFC Network Interactions

**Figure 4.**
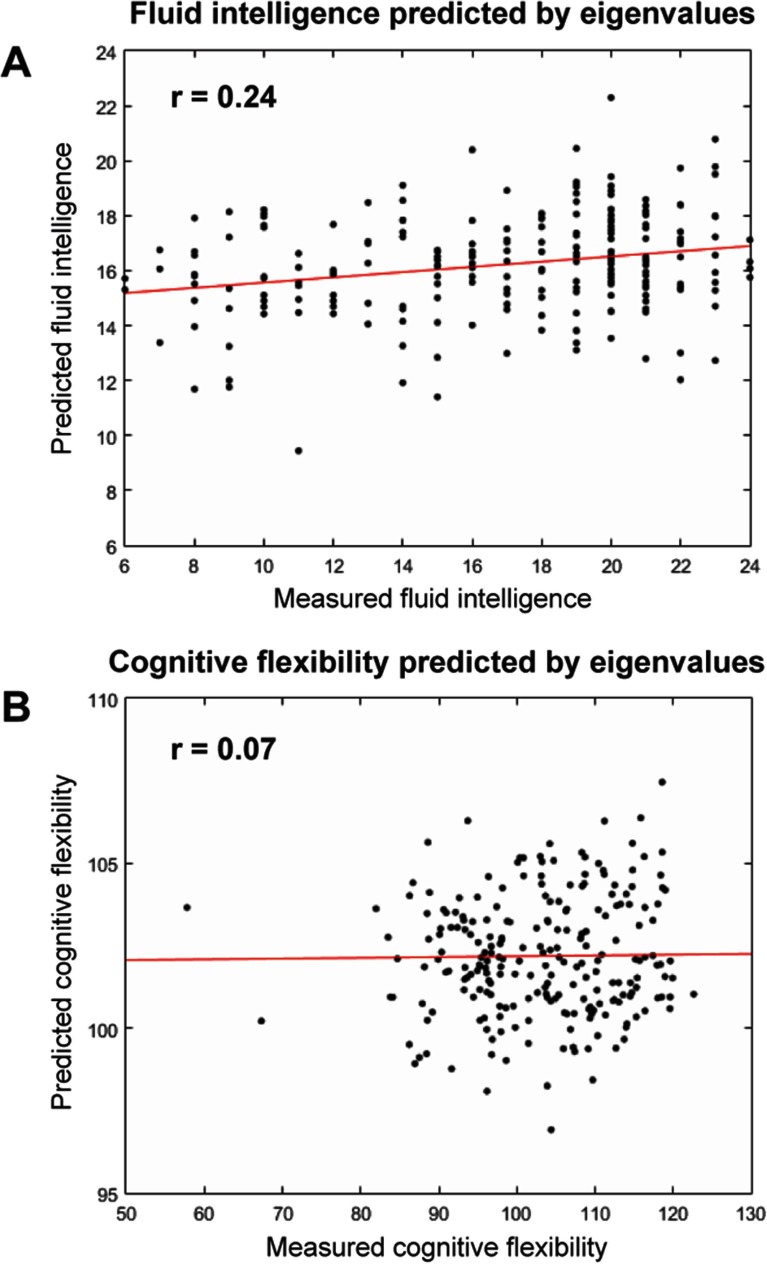
Predicting aspects of intelligence from resting-state functional connectivity A least-absolute-squares shrinkage operator (LASSO) regression trained in a subsample of *n* = 600 predicted fluid intelligence in an independent testing set (*n* = 230) with a correlation of *r* = 0.24 (*p* < 0.001; panel A). Using the same training set (*n* = 600) and testing set (*n* = 230) subsamples, LASSO regression predicted cognitive flexibility with a correlation of *r* = 0.07 (*p* = 0.28; panel B). The differential in predictive power between fluid intelligence and cognitive flexibility indicates that the spectral features associated with fluid intelligence represent more unique cognitive variance than do the spectral features associated with cognitive flexibility.

Using a LASSO regression determined from a training set of 600 participants, 33 of the 256 possible unique combinations of network interactions were used as predictors for fluid intelligence in an independent testing set (*n* = 230). The motivation for identifying combinations of networks related to intelligence is the hypothesis that individual differences in intellectual abilities may be represented better by the interactions of functional networks. A correlation score of *r* = 0.24 (*p* < .001) between the measured and predicted values of fluid intelligence was observed in the testing set (Figure 4A). For cognitive flexibility, 48 of the 1,024 possible unique combinations of network interactions were selected by LASSO regression as the best-fit model of cognitive flexibility from the PC eigenvalues. A correlation score of *r* = 0.07 (*p* = .28) was observed between the predicted and measured scores of cognitive flexibility (Figure 4B). However, predictions for cognitive flexibility based on eigenvalue products failed to reach significance, indicating that the eigenvalue magnitude for PC 1—that is, the strength of global synchrony—is a better predictor of cognitive flexibility than are models based on network interactions. Together, these results suggest that fluid intelligence and cognitive flexibility are associated with greater network stability or synchrony for the networks related to PCs 3–10. In contrast, cognitive flexibility, but not fluid intelligence, is negatively associated with overall synchrony, or stability, within the brain’s intrinsic functional architecture.

## DISCUSSION

We investigated how individual differences in the intrinsic network architecture of the brain are associated with human intelligence. Building from previous studies (Finn et al., 2015; Hearne et al., 2016; Smith et al., 2013), here we applied spectral decomposition methods to large-scale functional brain networks to determine whether spatially overlapping patterns of synchrony within these networks corresponded with two core aspects of intellectual functioning: fluid intelligence and cognitive flexibility. Both fluid intelligence and cognitive flexibility were reliably associated with the functional architecture of intrinsic brain networks. Synchrony within multiple functional networks reliably predicted fluid intelligence. Greater relative synchrony of networks within the network architecture (i.e., scaled eigenvalues) was associated with greater cognitive flexibility, whereas greater whole-brain baseline synchrony levels were associated with reduced flexibility. These results are consistent with our prediction that systems-level descriptors of the intrinsic functional architecture of the brain can provide reliable markers of human intellectual functioning.

Properties of the brain’s intrinsic neural architecture have been used to predict a range of cognitive capacities and may be a powerful predictor of more stable, or metacognitive, capacities such as intelligence (Stevens & Spreng, 2014). Previous reports examining the associations between resting-state functional connectivity and intelligence have identified pairs of nodes (i.e., network edges) that are predictive of cognitive ability using multiple regressions for feature selection (Finn et al., 2015; Hearne et al., 2016; Smith et al., 2013; Smith et al., 2015). Conceptually, our approach is consistent with these studies, in the sense that we characterize features of the brain’s functional architecture in order to identify large-scale distributed networks that support intellectual abilities. Our approach also builds upon these previous studies in several key ways. First, we identified whole-brain distributed networks using spectral decomposition applied to the resting-state connectome (Figure 1), rather than using pairwise correlation values as the predictive features for our model. We then used the singular values (i.e., eigenvalues) of the first ten principal components (eigenvectors), representing the persistence of within-network synchrony, to measure relationships between whole-network synchrony and intelligence (Figures 2 and 3). This method supports the findings from previous network approaches to identify the predictors of intelligence (Finn et al., 2015; Hearne et al., 2016) and positive (or negative) real-life functioning (Smith et al., 2015). The present study corroborates these earlier reports that whole-brain network connectivity and interactivity are biomarkers of fluid intelligence (Finn et al., 2015; Hearne et al., 2016; Smith et al., 2015) and supports the conclusion that its large-scale network architecture reflects individual differences in human intellectual functioning.

As we noted above, singular values for PCs are interpreted as reflecting the temporal stability of brain networks. In simulations iterating brain activity from a set of initial conditions based on functional connectivity between brain regions (Ferguson & Anderson, 2012), the PCs of functional connectivity matrices by definition correspond to patterns of activity that are invariant to change in a Markov model based on functional connectivity between regions. We predicted that more temporally stable patterns of brain activity would be associated with higher intelligence across a broad range of brain networks.

Consistent with this possibility, our results demonstrated that across-network interactions at rest are predictive of fluid intelligence more strongly than are correlations with the synchrony strength of any individual network (Figure 4). Cross-network interactions between the default network and frontoparietal networks have previously been reported to correspond positively with individual differences in fluid intelligence (Finn et al., 2015; Hearne et al., 2016). Our results are compatible with these findings of across-network interactions supporting fluid intelligence, and they extend this cross-network interaction paradigm for fluid intelligence to include a diverse set of resting-state functional networks (see Figures 1 and 4). Although our findings do not exclude a disproportionate effect of some connections, such as frontoparietal region pairs, which may have influenced many of the PCs in our analysis, the data suggest that additional information contributes to fluid intelligence in across-network synchrony.

As we demonstrated here, spectral decomposition of resting-state functional connectivity is consistent with a “one-to-many” functional architecture, with brain regions being implicated in multiple PCs, or networks. It is widely accepted that single brain regions can be recruited during multiple cognitive processes (McIntosh, 1999). One constraint of common decomposition methods such as spatial independence (Beckmann et al., 2005) or a greedy, winner-take-all algorithm (Yeo et al., 2011) is that they may obscure this one-to-many architecture. The PCA approach used here to identify patterns of functional synchrony (i.e., intrinsic brain networks) enables the identification of “promiscuous” brain regions that flexibly couple with multiple networks, providing the neural substrate for distributed parallel processing, a necessary condition for higher cognitive processes associated with intellectual functioning.

Structural research on neural contributions to intelligence has indicated that greater efficiency of physical connections within networks is positively correlated with individual differences in cognitive performance (Li et al., 2009; Pineda-Pardo et al., 2015). These findings may provide insight into the ways that individual differences in functional synchrony might arise from variations in anatomical connections. Integration of structural and functional data indicates that both efficiency and synchrony characterize intelligent brains (Pineda-Pardo et al., 2015). Greater efficiency in structural connectivity may directly contribute to more persistent synchrony within functional networks, in turn producing greater cognitive capacity. Future investigations, integrating multilayer maps that can incorporate functional synchrony, structural efficiency, and other physiological or genetic factors, will be necessary to identify truly systems-level biomarkers of human intelligence.

A major challenge in identifying neural makers of intellectual functioning has been the variability in research methodologies and in the operationalization of intelligence across studies (Sternberg, 2005). A prominent approach has involved the investigation of domain-specific processes associated with general intelligence, such as working memory capacity (Ackerman,et al., 2005; Conway et al., 2003; DeYoung et al., 2009; Edgin et al., 2010). However, mounting neural evidence is challenging such domain-specific conceptualizations of intelligence and supporting the existence of a more unitary or “multiple-demand” account (Duncan, 2010; Duncan et al., 2000) of fluid intellectual functioning, implicating the frontal parietal control network (Vincent et al., 2008). Meta-analyses of fMRI studies provide partial support for this idea. As was suggested by Duncan (2010), multiple brain regions are commonly recruited during cognitive control tasks associated with fluid intelligence (Basten, 2015). However, this multidemand network flexibly couples with other brain regions depending on the specific task context, including perceptual, mnemonic, or motor output demands (e.g., Niendam et al., 2012). This hybrid model, combining cognitive control and fluid intellectual functioning more broadly, is consistent with our findings, which also suggest that multiple brain networks (PCs 3–10) couple with core frontoparietal brain regions, yet remain differentiated by network-specific recruitment of brain regions outside the frontoparietal control network.

Patterns of persistent frontoparietal network synchrony are found in PCs 6, 7, and 10 and correspond to previous evidence that fluid intelligence, or “g,” may be preferentially dependent on the integrity of frontoparietal control networks and the underlying white-matter pathways (Penke et al., 2012, and see above). Cognitive control processes linked to fluid intelligence are necessary to form, reconfigure, or consolidate interpretive schema and are robustly linked with frontoparietal functional anatomy (Cole et al., 2015; Cole et al., 2013; Cole et al., 2012; Spreng et al., 2013). The positive correlations observed with the eigenvalues for these components suggest that fluid intelligence is supported by the cooperation of multiple spatially overlapping functional networks defined in our model, consistent with previous meta-analyses (e.g., Niendam et al., 2012). Furthermore, individuals with higher levels of intellectual functioning have greater brain “resilience,” such that those demonstrating more spatially distributed patterns of neural recruitment during cognitive tasks may be less susceptible to age-related brain changes or brain insult (Santarnecchi, Rossi, & Rossi, 2015). Our results here are also consistent with this idea, demonstrating that an intrinsic functional architecture comprising multiple, widely distributed brain networks, organized along a continuum of differentiated network synchrony, may provide the necessary neural foundation for the expression, and presumably the preservation, of fluid intelligence.

Interestingly, the magnitude of intrinsic global synchrony (PC 1) was negatively associated with cognitive flexibility (Figure 2). This may suggest, perhaps somewhat intuitively, that greater whole-brain synchrony—conceptually related to stability—is associated with lower mental flexibility. PC 1 is unique among the components in that it did not reflect some brain regions as positive and other brain regions as negative, so differences specific to PC 1 in our behavioral correlations may reflect global synchrony versus antagonistic or competing interactions between brain regions reflected in successive PCs. Cognitive flexibility, as such, appears functionally related to lower global synchrony and more network flexibility, a pattern of neural activity positively associated with learning and health (Bassett et al., 2011; Braun et al., 2015).

We suggest that a flexible network architecture with interacting brain networks is necessary for flexible thought and behavior, and may be a critical element of adaptive real-world functioning. Investigating this association between network and behavioral flexibility and how it changes as a result of aging and brain disease will represent an important area of future research. However, this observation also highlights the importance of studying functional brain networks, not simply in isolation, but also in relation to other networks, as well as studying within-subject variability in global network characteristics, as we did here. By scaling individual network synchrony values by each individual’s global network synchrony, we were able to identify robust correlations between these scaled eigenvalues and cognitive flexibility for all identified networks (PCs 2–10 in Figure 3).

Our findings suggesting that greater network synchrony is associated with higher fluid intelligence are consistent with reports that the brain network configuration at rest is closely aligned with task-driven network configurations in individuals of higher intelligence Schultz, 2016. These resting-state (or intrinsic) connections are hypothesized to reflect the persistence of task-based synchrony patterns formed through recurrent coactivation of distributed brain regions occurring over the course of development (Stevens & Spreng, 2014). These patterns of coactivation may serve as the brain’s “ready state,” potentiating task-driven activity among regions that commonly work in concert (Fair et al., 2007). The functional network organization associated with higher cognitive functioning may position individuals in an optimized “ready stance” that is prepared to engage cognitive tasks. However, future research will be necessary to more directly investigate this relationship.

These measures describe spectral components of RSFC as novel features of the brain’s functional architecture. Here we have shown that individual differences in intrinsic connectivity predict core aspects of human intelligence. Mapping intelligence in the human brain with RSFC offers a novel approach to investigating psychological functioning in health and disease. Alterations in the absolute and relative prominences of networks within the brain’s intrinsic functional architecture may help predict individual differences in normal cognitive functioning, as was demonstrated here, or predict patterns of altered cognitive abilities in neurodevelopment, normal aging, and brain disease.

## METHODS

### Resting-State Functional Connectivity

The Human Connectome Project (HCP) is an initiative by the National Institutes of Health to generate large, open-access behavioral and fMRI datasets. RSFC was preprocessed and analyzed for 830 subjects (mean age = 28.8 years, *SD* = 3.9, range = 22–37; 465 women) from the Human Connectome Project (www.humanconnectome.org; HCP900 release). The subjects were selected on the basis of having four complete resting-state scans and complete behavioral metrics for the cognitive features of interest. The BOLD fMRI data were acquired in four 15-min blocks. For the analysis, we used data cleaned using the FIX software (Glasser et al., 2013; Griffanti et al., 2014; Moeller et al., 2010; Setsompop et al., 2012; Van Essen et al., 2013).

The cerebral cortex was parcellated into 333 functionally defined regions (Gordon et al., 2014). Fourteen subject-specific subcortical regions were added using Freesurfer-derived segmentation (Fischl et al., 2002) of bilateral thalamus, caudate, putamen, amygdala, hippocampus, pallidum, and nucleus accumbens. Fourteen cerebellar regions were also added (Buckner et al., 2011). This combined parcellation scheme covering the full cortex, subcortical structures, and the cerebellum comprised a total of 361 regions. BOLD time series for each region of interest (ROI) were extracted, and Fisher’s *r*-to-*z* transformed Pearson correlation coefficients were obtained for each pair of ROIs in each 15-min block for each subject. The resulting 361 × 361 matrices were averaged across the four blocks for each subject and subsequently averaged across all subjects to obtain a group-level functional connectivity matrix.

Spectral decomposition of RSFC data produces functionally orthogonal principal components (PCs). These components are synonymous with eigenvectors of RSFC matrices and identify covariance patterns in the functional brain data. As such, PCs from group-mean and single-subject RSFC matrices represent a set of hierarchically organized intrinsic brain networks, ranked by the amount of signal variance described by each component. A functional network thus defined is a set of multivariate patterns that explain persistent synchrony between distributed brain regions during rest. In addition to identifying RSFC networks, spectral decomposition also calculates a singular value, alternatively called an *eigenvalue*, for each principal component vector. These are single numerical scores for the principal-component vector length and are proportional to the amount of total variance in the RSFC matrix accounted for by that component.

Principal components were identified using singular value decomposition of the 361 × 361 functional connectivity matrices (corresponding to the cortical-and-subcortical parcellation scheme; see above). The first ten PCs were calculated from the group mean connectivity matrix and back-projected onto anatomical space (Figure 1A). The first 20 PCs were identified in the same manner for each subject. Spatial correlations between the individual and group PCs were calculated using Pearson correlations. The single-subject PCs with the highest spatial correlation to group PCs 1–10 were determined, in order to assess the homogeneity of the single-subject PCs across the population (Figure 1B). The homogeneity of PCs is an indicator of network stability and replicability across the study population. We selected the most stable, or homogeneous, PCs in our sample for the subsequent brain and behavioral analyses—that is, PCs 1 to 10. The reliability of the PC network architecture across the group was assessed by one-sample *t* tests (Figure 1A), family-wise error-corrected for multiple comparisons (*p* < 0.05).

Network synchrony, the core measure of network architecture in the present study, is represented by the eigenvalue of each PC. We also calculate a scaled eigenvalue for each PC, representing the relative dominance of that network within the network hierarchy. All scaled eigenvalues represent the connectivity strength of that network relative to the dominant component (PC 1). PCs with higher eigenvalues (and scaled eigenvalues) are more prominent within an individual’s resting-state network architecture. Greater prominence suggests that the regions that compose a functional network demonstrate more persistently synchronous activity across the resting-state time series and explain more of the variance of temporal fluctuations than do lower-ranked networks. PC 1 constitutes the highest-ranked component and is referred to as the *global synchrony component*. As we noted above, all scaled eigenvalues are scaled relative to this global synchrony component and reflect individual differences in the organization, or relative strengths, of networks within the overall network hierarchy.

### Behavioral Data

We used the HCP behavioral measures related to cognition for our analysis. The battery includes measures of cognitive flexibility (Gershon et al., 2013), continuous performance (Gur et al., 2001), delay discounting (Estle, Green, Myerson, & Holt, 2006), executive inhibition (Gershon et al., 2013), working memory (Gershon et al., 2013), episodic memory (Gershon et al., 2013), language comprehension (Gershon et al., 2013), processing speed (Gershon et al., 2013), fluid intelligence (Bilker et al., 2012), language decoding (Gershon et al., 2013), spatial orientation (Gur et al., 2001), and verbal memory (Gur et al., 2001). These HCP behavioral measures largely consist of tests developed and validated for the NIH Toolbox (Gershon et al., 2013). A full description of these measures, including assessment protocols, normative data samples, and psychometric properties, may be found at www.healthmeasures.net/explore-measurement-systems/nih-toolbox). Additional measures were included in the HCP behavioral battery to further explore the dimensions of human social, emotional, and cognitive function. For full data on these additional measures, please see www.humanconnectome.org; HCP900 release. Behavioral data were collected from HCP partner sites, including Washington University and the University of Minnesota.

### Behavior/RSFC Eigenvalue and Scaled-Eigenvalue Correlations

To investigate whether individual differences in resting-state functional synchrony reliably predicted intellectual functioning, we divided the available 830 participants’ data into two sets: a training set (*n* = 600) and a testing set (*n* = 230). Spearman correlation coefficients were calculated between the individual subjects’ scores for behavior and the eigenvalues for PCs 1–10 in the training set (*n* = 600, Figure 2) to allow for nonnormal distributions in the behavior scores. As described above, we calculated the scaled eigenvalue, based on each individual’s global synchrony component, for each network component by dividing all the eigenvalues in an individual’s resting-state network profile by the eigenvalue of PC 1. The scaled eigenvalues for PCs 2–10 were correlated with the cognitive measures using Spearman correlation (*n* = 600, Figure 3). We report all correlations with 95% confidence intervals that did not include zero and with *p*-values that survived multiple-comparison correction (Figure 3).

### Behavior/RSFC Network Interactions

Given our overall prediction that human intelligence may be best reflected in the dynamic network architecture of the brain, we hypothesized that fluid intelligence and cognitive flexibility may be more strongly associated with dynamic network interactions than with synchrony simply within specific networks. As such, we undertook a secondary brain and behavioral analysis, using the products of individual eigenvalues, rather than a linear model, to predict cognitive performance.

We used a least-absolute-squares shrinkage operator (LASSO) with the training set (*n* = 600) to optimize the selection of network interactions—that is, the eigenvalue product terms—to be used in the predictor model. The designation of 600 participants in the training set and 230 participants in the testing set was based on a 70%–30% partition scheme for machine-learning predictions (Weinberger, Blitzer, & Saul, 2005). We evaluated all possible combinations of networks for the RSFC principal components that correlated significantly with the cognitive measures (see above). Eight significant PC features met these criteria for fluid intelligence (Figure 2), and ten significant PC features met these criteria for cognitive flexibility (Figures 2 and 3). As such, there were 256 possible unique combinations of network interactions for networks covarying with fluid-intelligence features (2^8^), and 1,024 possible combinations of network interactions for networks covarying with cognitive-flexibility features (2^10^). LASSO regression (MATLAB, version 2012b, statistical toolbox) optimized the data-fitting between eigenvalue products and fluid-intelligence scores in the training set (*n* = 600) to 33 unique combinations of network interactions that could be used as predictor terms. Coefficients and intercepts from the training set for these 33 network interactions were used as predictors in the independent testing set (*n* = 230). Of the 1,024 possible network interactions between the cognitive-flexibility RSFC features, LASSO regression optimized the data-fitting in the training set to 48 unique combinations of network interactions that could be used as predictor terms. The coefficients and intercepts for these interactions were used to calculate predicted values for fluid intelligence and cognitive flexibility in the independent testing set (*n* = 230). The volume of an abstract, *n*-dimensional eigensubspace (a.k.a., a “hypervolume”) may be determined by the products of orthogonal eigenvector magnitude—that is, by multiplying eigenvalues. Ultimately, each of the various eigensubspace terms identified by LASSO may prove to represent a discrete, dissociable neurobiological contribution to the emergent phenomenon of general intelligence.

## ACKNOWLEDGMENTS

Data were provided by the Human Connectome Project, WU–Minn Consortium (PIs: David Van Essen and Kamil Ugurbil; Grant No. 1U54MH091657), funded by the 16 NIH institutes and centers that support the NIH Blueprint for Neuroscience Research; and by the McDonnell Center for Systems Neuroscience at Washington University. Support for the analysis was provided by the National Institute of Mental Health (Grant No. K08 MH092697) and the Alzheimer’s Association (Grant No. NIRG-14-320049) to R.N.S. We are grateful to Elizabeth DuPre, Karen Spreng, and Gary R. Turner for helpful advice and discussion in the writing of the manuscript.

## AUTHOR CONTRIBUTIONS

Michael A. Ferguson: Conceptualization; Data curation; Formal analysis; Investigation; Methodology; Software; Visualization; Writing – original draft Jeffrey S. Anderson: Data curation; Formal analysis; Methodology; Writing – review & editing R. Nathan Spreng: Conceptualization; Methodology; Supervision; Writing – review & editing

## TECHNICAL TERMS

Spectral decompositions: Operations that determine the orthogonal basis set of a matrix, e.g., eigendecomposition, singular-value decomposition, principal-component analysis
Resting-state functional connectivity: An fMRI method for mapping correlated patterns in spontaneous blood oxygen level dependent signal across the brain during wakeful rest
Basis set: Functions, represented as vectors, whose combinations describe a system in terms of its computational and theoretical components
Intrinsic network architecture: Large-scale patterns of resting-state functional connectivity across distributed anatomical regions
Fluid intelligence: A component of general intelligence that operationalizes pattern recognition and completion in cognitive testing
Cognitive flexibility: The mental ability to switch between different systems of representation, operationalized by the Dimensional Change Card Sort Task
Persistent synchrony: Sustained functional covariance of brain regions, or nodes, within a network
Eigenvalue: A singular-value representation for the amount of system variance explained by the corresponding eigenvector within a basis set of functions
Least absolute squares shrinkage operator (LASSO): A statistical learning method for selecting predictive features from large sets of independent variables through iterative regression
Connectome: Graph theory model for connections across the brain; the connectome can be represented as layers of spatially overlapping functional networks
